# Effects of simultaneous transcutaneous auricular vagus nerve stimulation and high-definition transcranial direct current stimulation on disorders of consciousness: a study protocol

**DOI:** 10.3389/fneur.2023.1165145

**Published:** 2023-08-24

**Authors:** Yutong Zhuang, Weihang Zhai, Qinghua Li, Haoyang Jiao, Qianqian Ge, Peijing Rong, Jianghong He

**Affiliations:** ^1^Department of Neurosurgery, Beijing Tiantan Hospital, Capital Medical University, Beijing, China; ^2^Department of Neurosurgery, The Second Clinical College of Southern Medical University, Guangzhou, China; ^3^Institute of Acupuncture and Moxibustion, China Academy of Chinese Medical Sciences, Beijing, China; ^4^College of Anesthesiology, Shanxi Medical University, Taiyuan, China; ^5^Institute of Documentation, Chinese Academy of Traditional Chinese Medicine, Beijing, China

**Keywords:** disorders of consciousness, high-definition transcranial direct current stimulation, transcutaneous auricular vagus nerve stimulation, simultaneous stimulation, EEG microstate

## Abstract

**Background:**

Non-invasive brain stimulation (NIBS) techniques are now widely used in patients with disorders of consciousness (DOC) for accelerating their recovery of consciousness, especially minimally conscious state (MCS). However, the effectiveness of single NIBS techniques for consciousness rehabilitation needs further improvement. In this regard, we propose to enhance from bottom to top the thalamic–cortical connection by using transcutaneous auricular vagus nerve stimulation (taVNS) and increase from top to bottom cortical-cortical connections using simultaneous high-definition transcranial direct current stimulation (HD-tDCS) to reproduce the network of consciousness.

**Methods/design:**

The study will investigate the effect and safety of simultaneous joint stimulation (SJS) of taVNS and HD-tDCS for the recovery of consciousness. We will enroll 84 MCS patients and randomize them into two groups: a single stimulation group (taVNS and HD-tDCS) and a combined stimulation group (SJS and sham stimulation). All patients will undergo a 4-week treatment. The primary outcome will be assessed using the coma recovery scale-revised (CRS-R) at four time points to quantify the effect of treatment: before treatment (T0), after 1 week of treatment (T1), after 2 weeks of treatment (T2), and after 4 weeks of treatment (T3). At the same time, nociception coma scale-revised (NCS-R) and adverse effects (AEs) will be collected to verify the safety of the treatment. The secondary outcome will involve an analysis of electroencephalogram (EEG) microstates to assess the response mechanisms of dynamic brain networks to SJS. Additionally, CRS-R and AEs will continue to be obtained for a 3-month follow-up (T4) after the end of the treatment.

**Discussion:**

This study protocol aims to innovatively develop a full-time and multi-brain region combined neuromodulation paradigm based on the mesocircuit model to steadily promote consciousness recovery by restoring thalamocortical and cortical-cortical interconnections.

## Introduction

Disorder of consciousness (DOC) is caused mostly by disruption of thalamocortical and cortical-cortical connections due to extensive destruction of long-range white matter fiber tracts after severe brain injury (1). Typically, patients with DOC are classified into two levels of consciousness according to CRS-R: vegetative state/unresponsive wakefulness syndrome (VS/UWS), comprising those who retain only basic brainstem reflexes and sleep-wake cycles but no purposeful behavior, and minimally conscious state (MCS), comprising those who have fluctuating but reproducible signs of consciousness such as movement to command, visual pursuit, and localization to noxious stimulation (2).

**Figure 1:**
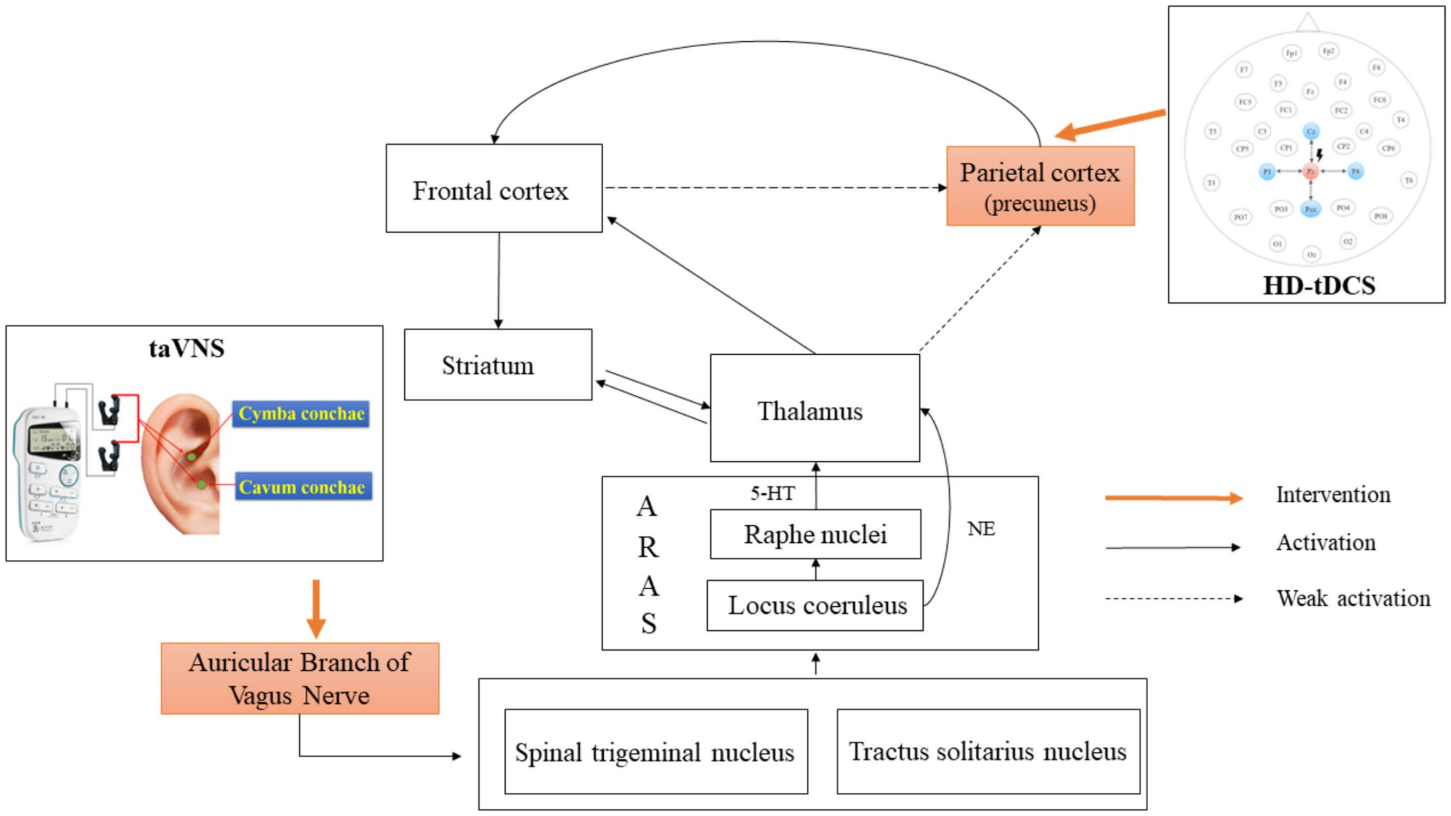
Mechanism of action of simultaneous taVNS and HD-tDCS. taVNS, transcutaneous auricular vagus nerve stimulation; HD-tDCS, high-definition transcranial direct current stimulation; 5-HT, 5-hydroxytryptamine pathway; NE, norepinephrine; ARAS, ascending reticular activating system.

In recent years, neuromodulation techniques have played an important role in treating neurological disorders through interventions on key hubs of the brain networks. taVNS is a novel NIBS technique that is safe and easy to use at home. It has been widely used and confirmed to have a good clinical effect on psychiatric disorders such as depression, insomnia, and cognitive disorders (3). In 2017, our team initially applied taVNS to patients with DOC and reported a case of a VS/UWS patient who improved to MCS after treatment (4). Briand et al. (5) further proposed a vagal cortical pathway model and suggested that taVNS might enhance the afferent signals from the auricular branch of the vagal nerve, promoting activity of the tractus solitarius nucleus and spinal trigeminal nucleus. Then, the neural impulses along the ascending reticular activating system (ARAS) strengthened from bottom to top the thalamic-striatal-cortical interaction to promote the recovery of consciousness (Figure 1) (5). Similarly, a subsequent longitudinal case study found another patient had improved from VS/UWS to MCS during taVNS treatment. However, the patient never fully regained consciousness after 6 months of continuous treatment and even had a downward trend in CRS-R after the end of treatment (6). Another study with a larger sample size reported that only 5 out of 14 DOC patients showed improvement in consciousness after treatment with taVNS (7). Similar results reported by Yu et al. (8) showed also that only 50% of patients with DOC had a good outcome after treatment. The fluctuation in the effective rate between study groups demonstrated that taVNS had a poor and unstable therapeutic effect on patients with DOC, which suggested a limited understanding of the underlying mechanism of taVNS for consciousness. That highlighted the urgent need for additional in-depth studies.

Yu et al. (8) further discovered a significant increase in cerebral blood flow in DOC patients with better prognoses after 1 month of taVNS treatment. The affected regions included the right thalamus, right caudate nucleus, left insula, superior temporal gyrus, left prefrontal cortex, precentral gyrus, and left occipital cortex. An EEG study also found that frontal–parietal and frontal-occipital connectivities were enhanced in MCS patients after 2 weeks of taVNS treatment (9). For the healthy subjects, a review pointed out that taVNS might commonly activate the thalamus, striatum, medial prefrontal cortex, and postcentral gyrus via the 5-hydroxytryptamine pathway of raphe nucleus and the norepinephrine pathway of locus coeruleus (5). In addition, studies using EEG have found increased power in lower frequency bands in healthy subjects after stimulation of taVNS, especially in the frontal and central regions (10). Based on the above evidence, we hypothesize that taVNS may initially activate the frontal regions at the cortical-cortical level. Then, these signals will disseminate to the parietal and occipital lobes through anatomical connections. However, the indirect weak effects via frontal cortex connectivity may not be enough to completely activate the posterior brain regions (Figure 1). According to the global workspace hypothesis, the formation and maintenance of consciousness required the integration of information from a large-scale frontoparietal network (11). Schiff et al. also proposed the mesocircuit model. They suggested that the initiation of consciousness relied on the interconnection via the thalamus between the frontoparietal network and anterior forebrain mesocircuit, which includes the frontal and prefrontal cortex and the striatopallidal negative feedback loop (1, 12). In conclusion, taVNS may modulate only a single neural circuit or local brain area, which cannot trigger the reconstruction of a complete consciousness network.

Transcranial direct current stimulation (tDCS) is another NIBS technique and is characterized by direct modulation of cortico-cortical connections in a top-down manner (13). Conventional tDCS consists of two electrode pads. The anode increases the excitability of the target area through subthreshold weak stimulation, while the cathode acts as an inhibitor (14). It is generally accepted that the selection of stimulation sites is a critical factor influencing the moderator effect of tDCS on neural networks (15–17). The precuneus and posterior cingulate cortex (PCC) are key nodes of the default mode network (DMN) in the posterior brain region. The precuneus/PCC and the posterior medial cortex were recognized as regions with the highest metabolic activity during the resting state in healthy subjects (18). In addition, the functional diversity and integration of the precuneus and PCC were significantly lower in patients with DOC than in healthy subjects (19). Another study further revealed that an increase in the metabolic ratio of the precuneus to the central thalamus was accompanied by improved levels of consciousness (20). Therefore, the activity of precuneus and PCC was correlated with the level of consciousness. Consequently, it was expected to be a potential stimulation site of tDCS in posterior brain regions to promote restoration of consciousness. A randomized, crossover, controlled trial found that nine patients with DOC showed behavioral recovery after repeated treatments of tDCS targeting the posterior parietal cortex (PPC) for 5 days. But the overall effective rate of the tDCS montages targeting the PPC was lower than the tDCS targeting the dorsolateral prefrontal (15). This disparity in effectiveness might be attributed to the diffuse current of tDCS that could not precisely and effectively stimulate the PPC. In this regard, HD-tDCS with a more focused stimulation current was developed to induce focal neural and specific behavioral changes. Guo et al. (21) used HD-tDCS targeting precuneus to treat patients with DOC and established that 72% (9/11) of patients with DOC had a significant increase in CRS-R scores. Furthermore, the simultaneous EEG results indicated a significant change in central-parietal connectivity, suggesting that HD-tDCS activated a wide range of brain activity outside the target (21). Thus, it seemed that HD-tDCS had a clear modulatory effect on the posterior brain regions of patients with DOC. But the overall effective rate of HD-tDCS for consciousness recovery was still precarious due to different stimulation paradigms in various studies (21–23).

In summary, we propose to enhance from bottom to top the overall activity of the anterior forebrain mesocircuit by using taVNS. Furthermore, HD-tDCS targeting precuneus will be used at the same time to compensatively enhance the frontoparietal network to overcome the issue that single NIBS techniques were insufficient to activate the large-scale consciousness circuit (Figure 1). Ultimately, the thalamocortical and frontoparietal network simultaneously will be maintained at a high level of excitability to restore the integrity of the consciousness network and accelerate the recovery of consciousness in patients with DOC. In the study, we will look into the clinical efficacy and safety of whole-time and multi-brain combined modulation to break the bottleneck of the unstable effect of NIBS techniques and deepen the understanding of the mechanisms of consciousness onset and maintenance in DOC patients.

## Methods

### Study design

**Figure 2:**
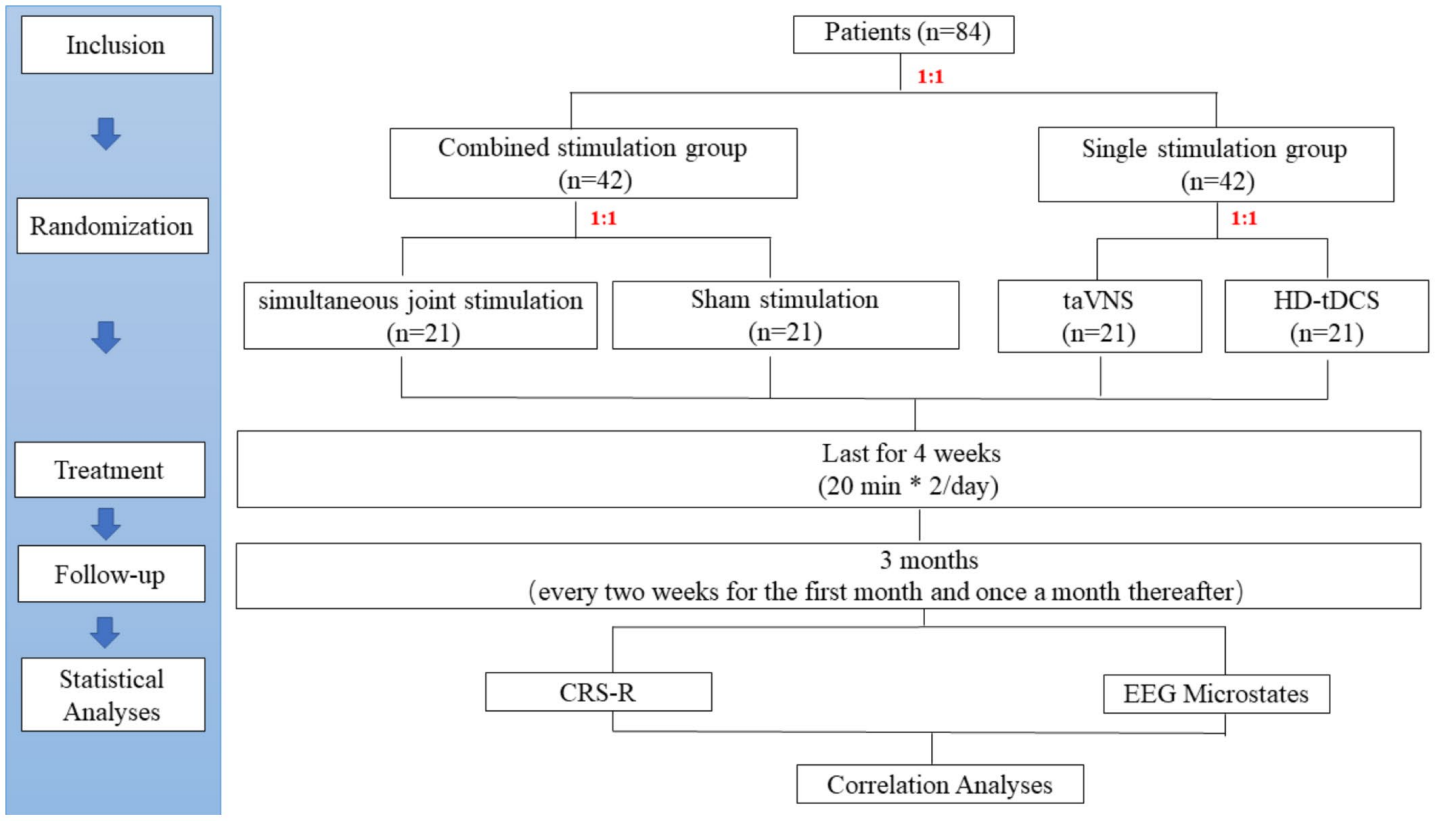
Study flow diagram. taVNS, transcutaneous auricular vagus nerve stimulation; HD-tDCS, high-definition transcranial direct current stimulation; NCS-R, nociception coma scale-revised; EEG, Electroencephalogram; CRS-R, coma recovery scale-revised.

The study is a prospective, randomized, controlled, double-blind clinical trial (Figure 2) that has been registered at the Chinese Clinical Trial Registry (ChiCTR2300069166). The study protocol is designed according to the Declaration of Helsinki and has been approved by the Ethics Committee of Beijing Tiantan Hospital, Capital Medical University (NO. KYSQ 2022-347-01). Informed consent will be obtained from a patient-authorized legal representative due to patients’ disorders of consciousness.

### Participants

The study has initiated in April 2023 and will continue until December 2024. A total of 84 MCS patients will be included in the Neurosurgery Inpatient Department of Beijing Tiantan Hospital. The inclusion and exclusion criteria are presented in Table 1.

**Table 1:** Inclusion and exclusion criteria.

**Inclusion**	
	1. Diagnosed with MCS by coma recovery scale-revised;
	2. Age: from 18 to 60 years;
	3. More than 1 month after the initial brain injury;
	4. Consciousness is in a stable phase (no change in the total score of CRS-R) for at least 2 weeks before admission;
	5. No cranial defect or extensive skull repair;
	6. Precuneus and posterior parietal should be intact on at least one side;
	7. Patient-authorized legal representative agreed to the experimental protocol and signed informed consent.
**Exclusion**	
	1. Neurodegenerative diseases such as Alzheimer’s disease and Lewy body dementia; 2. Disorders of consciousness caused by operation injuries or malignancy;
	3. Time since onset less than 1 month (acute coma);
	4. Patients with seizures that are difficult to control;
	5. Patients are undergoing other clinical trials;
	6. Pregnancy and lactation.
**Withdrawal**	
	1. Recurrent seizures during treatment;
	2. Life-threatening diseases such as severe lung infection, intracranial infections, and cerebral hernia;
	3. Death;
	4. Patient is lost to follow-up.

### Sample size

The sample size was calculated based on the effective rate. An improvement of at least 3 points in the CRS-R was considered an effective treatment. Previous studies found the effective rate of taVNS was 7% (*n* = 14) (7) and of HD-tDCS was 36% (*n* = 11) (21). Assuming the existence of a synergistic effect, the SJS group will have a higher effective rate of 43%, while the sham stimulation group will have no effect. Therefore, the two-sided 2 × 4 chi-square test in PASS (version 15) was used for the sample size calculation of four groups. The test power (1-β) was 80% and the type I error rate (α) was 5%. The calculated effect size W was 0.436, and then a dropout rate of 20% was considered. Finally, a total of 84 patients will eventually be enrolled, and each group will have 21 patients (Figure 2).

### Procedures

The randperm function of MATLAB (Version 2020b, MathWorks Inc., Natick, United States) will be used to generate 84 random integers in random order by the study leader. Each patient will be given a random number in the order of enrollment. The first randomization will divide patients into a combined stimulation group (1–42) and a single stimulation group (43–84) based on a 1:1 ratio. Patients in the combined stimulation group will be further randomized in a 1:1 ratio into the SJS group (1–21) and the sham stimulation group (22–42). Similarly, patients in the single stimulation group will also be randomly assigned in a 1:1 ratio to the taVNS group (43–63) and the HD-t DCS group (64–84).

The study protocol is a double-blind design. Each patient will be exposed to identical-looking simultaneous stimulation. Specifically, the SJS group will receive both positive stimulation of taVNS and HD-tDCS. In contrast, the sham stimulation group will receive sham stimulation of taVNS and HD-tDCS to eliminate potential brain effects caused by the physical compression of the instrument. The patients in the single stimulation group will receive one kind of positive stimulation by taVNS or HD-tDCS. They will also receive sham stimulation of another technique at the same time to blind patients/families and therapists/assessors.

All patients will receive treatment twice a day in the morning and afternoon for 4 weeks. There are 2 days’ rest every 5 days of treatment. Considering the high current intensity of SJS, the single stimulation time of taVNS, commonly for 30 min (24), will be adjusted downward to 20 min to reduce the burden on the patient’s brain.

The Ethics Committee of Beijing Tiantan Hospital will be responsible for independent security monitoring. Adverse events and unintended events will be reported to them when every 10 patients are included. They will further assess the causal relationship between the adverse reaction and treatment. If a serious adverse event is proven to have been caused by the treatment, the trial will be terminated immediately. Appropriate medical emergency and protective measures will be given to patients at the same time.

### Stimulations

The stimulation area of the taVNS (SDZ-IIB, Suzhou Medical Supplies Factory) will be bilateral auricles. A pair of clips will be placed on one side of the auricle and another pair of clips will be placed on the opposite side. A clip will have three carbon-impregnated silicone tips. The first tip will serve as the common end of the other two tips to support the posterior surface of the auricle. The second tip will be placed on the lateral scapha. The third tip will be placed on the medial auricular cavity to stimulate targets including cymba conchae and cavum conchae (Figure 1). The stimulator will provide electrical pulses of 1–1.5 mA with an alternate frequency between 4 and 20 Hz and a pulse width of 30 μs. Stimulus intensity will be routinely set at 1.5 mA for each patient. We will turn down the current when the patient’s blood oxygen saturation is below 95%, heart rate increases by more than 20%, or the NCS-R score exceeds 3 points and shows a significant increase during stimulation compared to pre-stimulation. The stimulator will have no ramp-up and ramp-off of current during the stimulation. In addition, two identical-looking instruments will be used. One, called number 1, will be capable of normal stimulation as positive stimulation after the switching on. However, the other one, called number 2, will have no current output as a sham stimulation.

**Figure 3:**
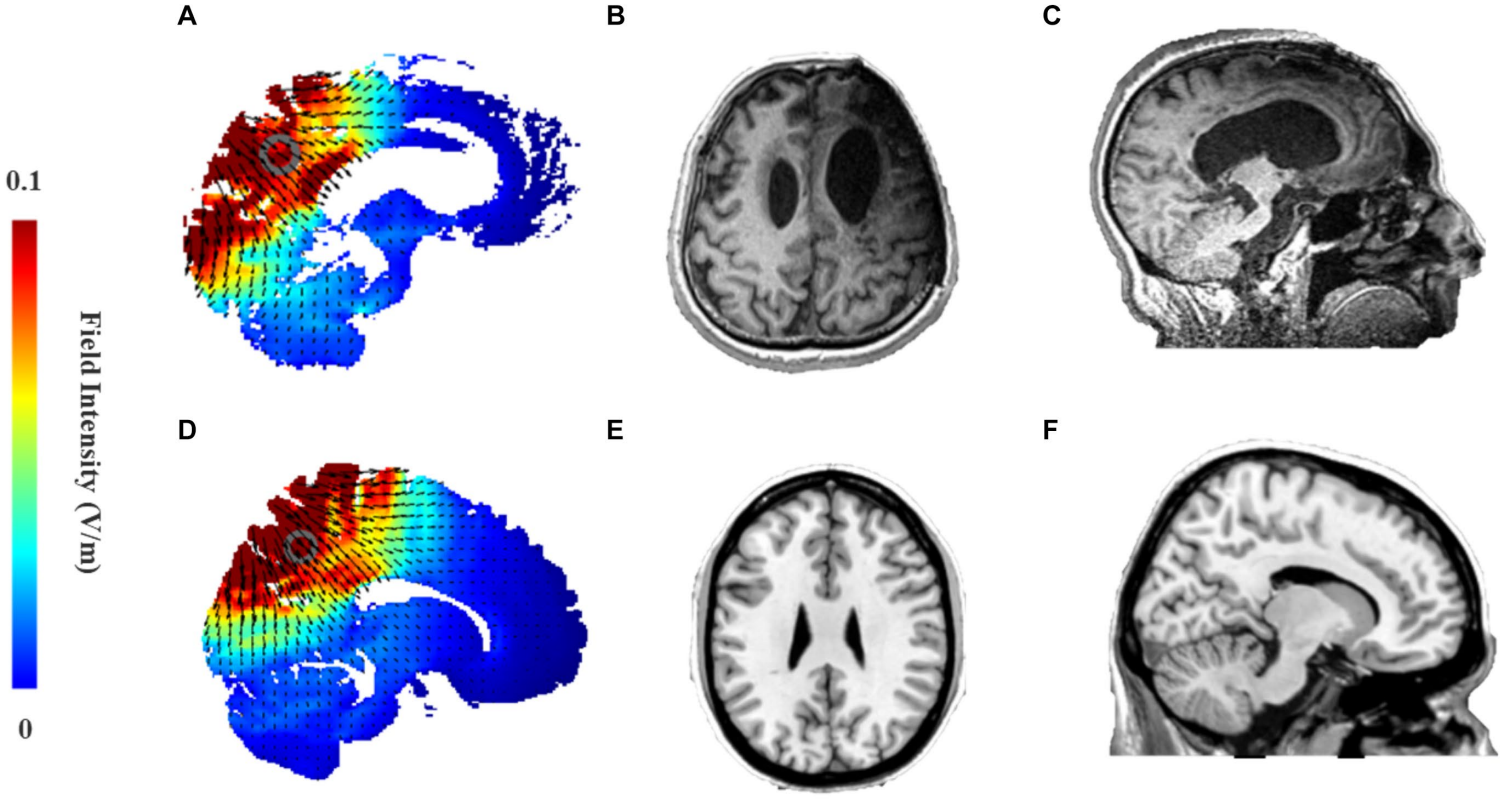
**(A,D)** Electric field intensity map in stimulation model from ROAST of 2.0 mA HD-tDCS that targets precuneus using Pz as the central stimulation electrode and Cz, P3, P4, POz as peripheral return electrodes. **(A)** Sagittal brain activation map of the MCS patient; **(D)** Sagittal brain activation map of Colin27 template; **(B,C)** Head MRI of an MCS patient caused by traumatic brain injury. **(B)** Axis plane; **(C)** Sagittal plan. **(E,F)** Head MRI of Colin27 standard template in the MRIcorn software. **(E)** Axis plane; **(F)** Sagittal plane.

The HD-tDCS (4 × 1-C2, Soterix Medical Inc.) stimulation target will be the precuneus. Pz according to an international standard 10–20 EEG system has been proven as a position overlying the medial parietal cortex and the precuneus (25). Therefore, the central electrode of HD-tDCS is placed at Pz, and the four return electrodes are placed at a distance of approximately 3.5 cm from the central electrode at Cz, P3, P4, and POz (Figure 1). The stimulation current is a constant 2 mA with a ramp-up time of 30 s and a ramp-down time of 30 s. According to simulation modeling from a realistic volumetric approach to simulate transcranial electric stimulation (ROAST) (26), the HD-tDCS montage can effectively activate the PPC and precuneus of patients with DOC (Figure 3A). In terms of sham stimulation of the HD-tDCS, its parameters will be the same as positive stimulation with the difference that the voltage will rise to 2 mA and decrease immediately to 0 mA when the instrument is turned on. The positive and sham stimulation start buttons will be, respectively, covered by two labels called numbers 3 and 4.

The therapist will be informed of the HD-tDCS button number and the taVNS machine number that should be used for the treatment of each patient by the study leader before treatment. However, the therapist will not know the specific function of each number.

### Neuroimaging assessments

T1-weighted brain images will be obtained using a 3.0 T magnetic resonance imaging (MRI) scanner (HD750, GE, United States) to evaluate the resting-state brain structure in each patient before enrollment. Patients with severe damage in the precuneus/PCC will not be included in the study to ensure the effectiveness of HD-tDCS stimulation.

### Behavioral assessments

**Figure 4:**
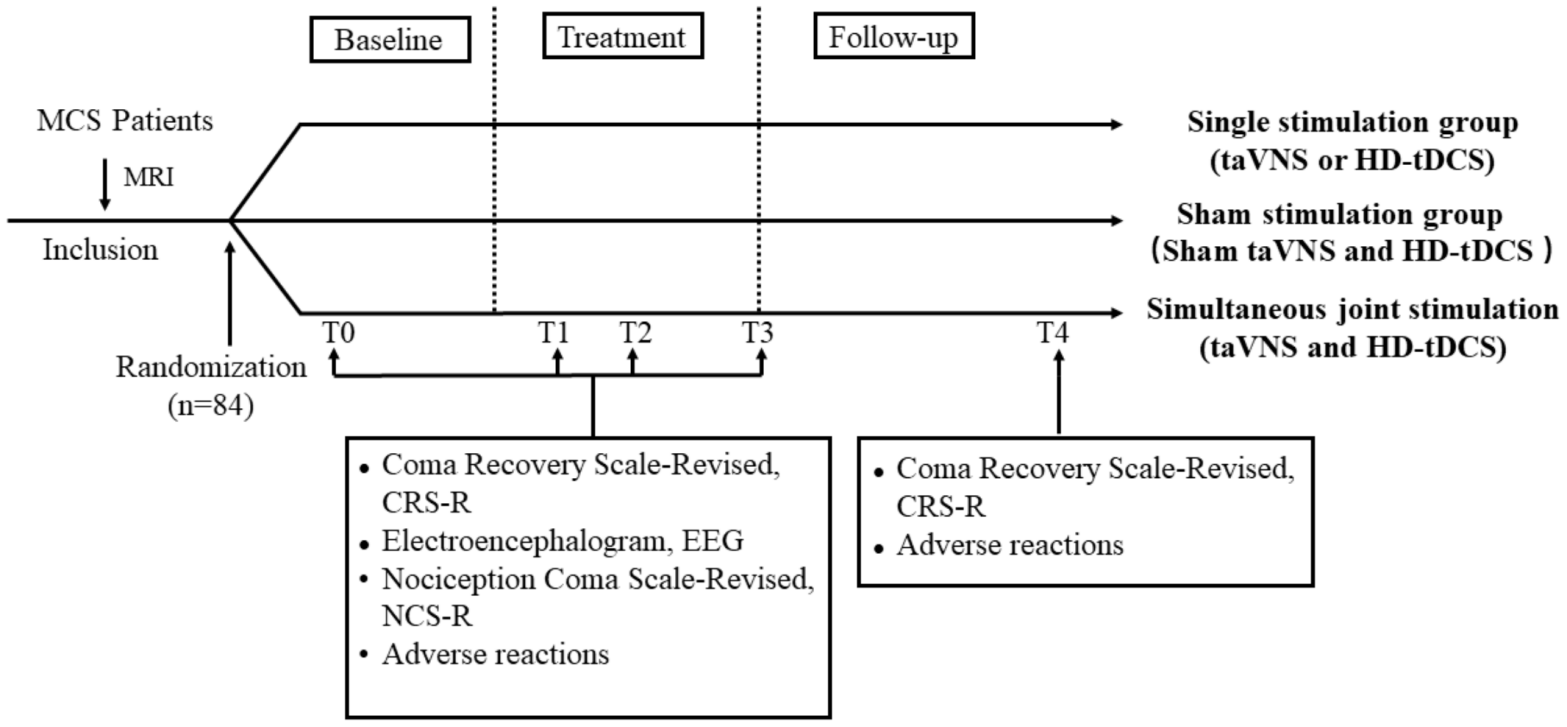
Treatment and data collection flow diagram. T0: 2 weeks before treatment; T1: 1 week of treatment; T2: 2 weeks of treatment; T3: 4 weeks of treatment; and T4: 3-month follow-up. MRI, magnetic resonance imaging; taVNS, transcutaneous auricular vagus nerve stimulation; HD-tDCS, high-definition transcranial direct current stimulation.

The CRS-R was first proposed by Giacino et al. (2) to assess the level of consciousness in patients. The scale with a total of 23 points included six subscales of auditory, visual, motor, verbal, communication, and arousal levels (2). In the study, patients will be evaluated independently by two trained clinicians. They will perform five repeated CRS-R assessments at least 2 weeks before enrollment to clarify the patient’s state of consciousness and clinical diagnosis. Finally, the highest CRS-R score will be taken as the pre-treatment (T0) baseline score. Changes in CRS-R will be assessed at three time points during treatment to reflect the effect of treatment: 1 week (T1), 2 weeks (T2), and 4 weeks (T3) (Figure 4).

Schnakers et al. (27) developed the nociception coma scale (NCS) to assess the nociception of patients with DOC, which included four subscales of motor response, verbal response, visual response, and facial expression (27). Subsequently, Chatelle et al. (28) excluded the visual subscale to propose a more sensitive new version called NCS-R (ranging from 0 to 9 points) for assessment of nociception compared to the NCS. They found that an NCR-R cut-off value of 4 points could differentiate MCS patients’ behaviors induced by nociceptive stimulation from behaviors induced by non-noxious stimulation (28). The NCS-R will be used before and during stimulation in the study to adjust the current intensity of taVNS.

### EEG recording and microstate analysis

The resting 30-min EEG signal will be recorded at a 1,000 Hz sampling rate by 32 Ag/AgCl electrodes (Nicolet EEG V32, Natus Neurology, United States) according to the international standard 10–20 EEG system (the detailed sites of 32 electrodes can be seen in Figure 1) at each time point. The impedance between the electrode and the patient’s skin will always be kept below 5 kΩ. EEG monitoring will be stopped when the patient is tired or asleep, and then the patient will be kept awake by stimulating the patient’s earlobe.

The EEG raw data will first be preprocessed offline in MATLAB (Version 2020b, MathWorks Inc., Natick, USA). The preprocessing will mainly consist of 2–20 Hz bandpass filters, 50 Hz notch filters, and down-sampling to 250 Hz. EMG and EEG artifacts will be removed by independent component analysis. Finally, all channels will be re-referenced to the average reference.

After the preprocessing, the 10-min noise-free EEG data will be imported into the Cartool toolbox^1^ for microstate analysis. Global Field Power (GFP) was the standard deviation of the amplitude at each point for all channels, which was used to characterize the instantaneous topographic field strength. When GFP was high, the topographic map maintained a relatively steady state and had a high signal-to-noise ratio (29). Therefore, the topographic maps corresponding to the GFP peak will be selected as the original maps for subsequent clustering analysis in this study. The clustering algorithm will be topographic atomize and agglomerative hierarchical clustering (30) with a range of clusters from 1 to 12. The optimal number of clusters will be determined by the meta-criterion (31). Subsequently, the spatial correlation coefficient between each original map and the microstate template maps will be calculated to determine the microstate category to which the original maps will belong. Then, the microstate time series will be smoothed with time frames set to 8 so that segments that are smaller than 30 ms will be rejected. In the end, four parameters of the EEG microstate will be calculated.

1. Duration: the average duration for which a microstate remains stable.
2. Occurrence: the mean occurrences of a microstate per second.
3. Coverage: the duration of a microstate divided by the total duration of all microstates.
4. Probability: the probability that a microstate transits to another microstate.

### Follow-up period

Each patient will be followed up for 3 months (T4) after the end of treatment to observe residual effects and delayed adverse effects. Follow-up visits will include outpatient visits, video calls, home visits, and surrogate assessments by other healthcare organizations. The follow-up will include an assessment of CRS-R and a recording of adverse effects. The frequency of follow-up will be every 2 weeks for the first month and once a month thereafter.

### Data safety and management

The medical history, demographic data, behavioral data, MRI data, and EEG data of all patients will be stored in the departmental computer database by the study leader. The Ethics Committee of Beijing Tiantan Hospital will regularly check data security.

### Statistical analysis

IBM SPSS Statistics 26 software will be used for statistical analysis. The measurement data will be tested for normal distribution by using Kolmogorov-Smirnov tests. Data conforming to a normal distribution will be analyzed by ANOVA or repeated measures ANOVA for differences in each treatment group. Data not conforming to a normal distribution will be analyzed by the Kruskal-Wallis H test or Friedman test. *Post hoc* tests will be carried out with Bonferroni. Count data will be expressed as cases or percentages, and differences between groups for count data with all theoretical frequencies greater than 5 will be tested by the chi-square test, otherwise, Fisher’s exact test will be used. *p* < 0.05 will be considered to indicate statistically significant differences.

## Discussion

With the advancement of technology, more and more NIBS techniques are being developed and applied in the treatment of patients with DOC, and are divided into two main categories according to the different targets. One is top-down NIBS techniques such as tDCS and repetitive transcranial magnetic stimulation (rTMS). They regulate cortical activity levels via a cortico-thalamo-cortical feedback loop (32). The other is bottom-up NIBS techniques, including taVNS and ultrasound deep brain stimulation. They intervene directly in the thalamus, and signals are further projected along ascending fibers to extensive cortical regions (8, 33). However, these single NIBS techniques of the treatment mechanisms and modulation paradigms are still being explored. Therefore, the overall effective rate is still low (34, 35).

In response, many researchers have begun to experiment with single-technique multi-targeted combined stimulation or multiple-technique combined stimulation modulation paradigms (17, 36). A study reported that an MCS patient showed object recognition, movement to command, and significant non-functional communication after 2 weeks of SJS of tDCS and rTMS targeting inferior parietal lobes (IPL). The patient’s CRS-R improved from 9 to 19 at the end of the follow-up. Meanwhile, they found that the improvement in CRS-R was accompanied by increased activities of IPL and PCC and improved connectivity in posterior brain regions (37). In addition, another study in 2021 reported that 30 healthy subjects had significantly stronger activation in the bilateral thalamus, pallidum, parahippocampal gyrus, dorsal raphe nucleus, and substantia nigra after SJS of taVNS and tDCS compared to any single stimulation, suggesting a significant synergistic effect (38). Thus, combined NIBS techniques are expected to break the upper limit of the effect of single modulation techniques.

The frontoparietal network, which is closely related to the recovery of consciousness, is composed mainly of two subnetworks: the executive control network (ECN) and the DMN (1). There is extensive competition between the two networks, which is necessary for flexible switching of attention. The ECN is responsible for external perception tasks. In contrast, the DMN is mainly involved in internal attention-directed cognitive processing such as autobiographical recall, imagining the future, and planning. Key nodes of DMN are divided into the medial prefrontal cortex in the anterior brain region and the precuneus/PCC and the inferior parietal lobe in the posterior brain region. It was reported that internal connectivity in DMN was significantly lower in VS/UWS than in MCS (39). Further study of the transient analysis found that disrupted functional connectivity in the alpha band of the anterior state between PCC and medial prefrontal cortex was accompanied by decreased level of consciousness, while high functional connectivity between the two regions indicated a positive prognosis in the distant future (40, 41). Thus, the interconnection of the two key anterior and posterior nodes within the DMN plays a key role in the generation of consciousness.

The precuneus is part of the superior parietal lobe and is located in the medial cerebral hemisphere. As mentioned in the introduction, it is hard for tDCS with low spatial resolution to focus current into the deep cortex. A recent study showed that HD-tDCS over Pz was able to activate the precuneus/PCC to improve memory retrieval performance in healthy subjects (42). As for patients with DOC, severe brain injuries often led to extensive damage and deformation of brain structures. The anterior forebrain mesocircuit was shown to be vulnerable to multifocal brain injuries due to widespread anatomical connections, while the posterior brain regions were shown to be more likely to be well-preserved (1, 21). To clarify the depth of stimulation of HD-tDCS over Pz in patients with DOC, we enrolled an MCS patient. His left frontotemporal lobe was severely damaged (Figure 3B), but there was only slight atrophy in the posterior brain regions compared to healthy subjects (Figures 3C,F). Then, his simulation modeling from ROAST demonstrated that 2 mA HD-tDCS could still effectively activate the less damaged PPC and precuneus (Figure 3A). Most studies have also generally found that HD-tDCS over Pz can effectively enhance information processing in posterior brain regions to promote recovery of consciousness (43, 44).

Based on the aforementioned theories and evidence from previous clinical studies, we propose to use taVNS to increase the excitability of the anterior forebrain mesocircuit and utilize HD-tDCS to strengthen the activities in the precuneus to facilitate the reconstruction of the frontoparietal network. Finally, SJS of taVNS and HD-tDCS will help the cortical and subcortical networks interconnect with the thalamus as the hub to reproduce the complete consciousness network.

The protocol will include only MCS patients because they have better neural plasticity and benefit more from taVNS and HD-tDCS compared to VS/UWS (9, 44). In addition, the duration of treatment is a key factor influencing the outcome. A meta-analysis summarized eight clinical studies of tDCS for DOC and found better outcomes in patients who received more than five repeated tDCS sessions than those who received only a single stimulation session (45). Wang et al. (9) used taVNS in 12 patients with DOC and none of them showed behavioral improvement after 2 weeks of treatment (9). In contrast, another study using a similar stimulation protocol but a longer stimulation time found that five patients regained consciousness, which might be due to the long-term potentiation of taVNS to increase excitatory synaptic connections (8). Therefore, 1 month was set as the treatment cycle in the study. For the primary outcome, we hypothesize that the SJS group will show a significant CRS-R increase after 2 weeks of treatment. This increase will be expected to reach its peak at 4 weeks post-treatment and remain during the 3-month follow-up. In contrast, the single stimulation group will show a significant increase in CRS-R only after 4 weeks of treatment compared to baseline. As for AEs, although tDCS and taVNS caused some mild or transient AEs, neither of them induced serious AEs. A review pointed out that the most common AEs of tDCS in patients with stroke were itching, burning sensation, headache, tingling, sleepiness, difficulty in concentration, mild fatigue, skin redness, and dizziness. Likewise, the most common AEs of taVNS were ear pain, headache, tingling, dizziness, skin redness, fatigue, prickling, pressure, itching, and unpleasant feeling (46–48). In this study, we will judge pain AEs by the NCS-R and observe bedside changes in patients’ ears, scalp, expressions, and levels of arousal and sleep to detect their discomfort and fatigue because patients will be unable to subjectively report their symptoms. In addition, if patients exhibit great residual motor function (motor subscale of CRS-R ≥ 3), we will determine the areas of pain induced by stimulation according to the patient’s performance on localization to noxious stimulation.

EEG was particularly suitable for bedside assessment of NIBS techniques in DOC because of its high temporal resolution and simplicity of operation. Microstate was a reliable method to assess dynamical changes of large-scale organized brain activity by clustering EEG topography (49). Most studies reported that four typical microstates (A–D) were sufficient to explain EEG resting activity. A microstate always remained relatively stable for 80–120 ms, and then it rapidly switched to another. The activity characteristic of the microstate was similar to the transient and metastable brain activation pattern of conscious activity (50). In the diagnosis of consciousness, microstate D was the best to classify VS/UWS and MCS patients among multiple quantitative indicators of resting-state EEG (51). Similarly, a study found that microstate D was more frequent in MCS compared to VS/UWS and was positively correlated with CRS-R. Further treatment of DOC by HD-tDCS for 2 weeks increased the frequency, duration, and coverage of microstate D in responders, while the duration and coverage of microstate C decreased (22) compared to the baseline. The latest study combined microstate C related to the salient networks and DMN (52) and microstate D related to the ECN as the L-R diagram. On the contrary, microstate A was related to the auditory network, and microstate B was related to the visual network, and both microstates were combined as the A-P diagram. It was found that the shorter duration of L-R diagrams and the higher incidence of A-P diagrams might reflect the higher-level language processing capacity of the brain in MCS (53). Therefore, microstate temporal dynamics features were reliable metrics to reflect the residual dynamic conscious activity of patients with DOC at the whole brain level. Microstates C and D might be associated with higher cognitive processing activities.

In conclusion, we intend to utilize combined stimulation to activate the intact consciousness loop and investigate the efficacy and safety of the new stimulation paradigm for speeding up the recovery of consciousness in patients with DOC. The protocol will additionally use EEG microstates that reflect global transient neural activity to evaluate the intervention mechanism of SJS on the dynamic activity of thalamocortical and cortical-cortical neural networks. These findings, in combination with clinical outcomes, will guide the subsequent development and optimization of the stimulation paradigm of combined modulation for multiple brain regions. The ultimate goal will be to achieve stable improvement in consciousness and confirm the importance of frontoparietal high-level connectivity for the formation and recovery of consciousness.

## Ethics statement

The study protocol involving humans was approved by The Ethics Committee of Beijing Tiantan Hospital. The protocol will be conducted in accordance with the local legislation and institutional requirements. The patient-authorized legal representative will provide their written informed consent to participate in this study.

## Author contributions

JH: conceptualization. JH and PR: funding acquisition. YZ and WZ: methodology and writing. QL: validation. HJ and QG: visualization and revision. All authors contributed to the article and approved the submitted version.

## Funding

The work was supported by the National Natural Science Foundation of China [No. 82272118 and 81771128] and the CACMS Innovation Fund [No. CI2021A03305].

## Conflict of interest

The authors declare that the research will be conducted in the absence of any commercial or financial relationships that will be construed as a potential conflict of interest.

## Publisher’s note

All claims expressed in this article are solely those of the authors and do not necessarily represent those of their affiliated organizations, or those of the publisher, the editors and the reviewers. Any product that may be evaluated in this article, or claim that may be made by its manufacturer, is not guaranteed or endorsed by the publisher.

## Footnotes

^1^https://sites.google.com/site/cartoolcommunity

## Glossary

DOC: Disorders of consciousness
NIBS: Non-invasive brain stimulation
MCS: Minimally conscious state
taVNS: Transcutaneous auricular vagus nerve stimulation
HD-tDCS: High-definition transcranial direct current stimulation
CRS-R: Coma recovery scale-revised
NCS: Nociception coma scale
NCS-R: Nociception coma scale-revised
EEG: Electroencephalogram
SJS: Simultaneous joint stimulation
ARAS: Ascending reticular activating system
VS/UWS: Vegetative state/unresponsive wakefulness syndrome
MRI: Magnetic resonance imaging
ECN: Executive control network
DMN: Default mode network
rTMS: Repetitive transcranial magnetic stimulation
PPC: Posterior parietal cortex
PCC: Posterior cingulate gyrus
ROAST: Realistic volumetric approach to simulate transcranial electric stimulation

## References

[1] Edlow BL Claassen J Schiff ND Greer DM. Recovery from disorders of consciousness: mechanisms, prognosis and emerging therapies. Nat Rev Neurol. (2021) 17:135–56. doi: 10.1038/s41582-020-00428-x, PMID: 33318675 PMC7734616

[2] Giacino JT Kalmar K Whyte J. The JFK coma recovery scale-revised: measurement characteristics and diagnostic utility. Arch Phys Med Rehabil. (2004) 85:2020–9. doi: 10.1016/j.apmr.2004.02.033, PMID: 15605342

[3] Wang L Wang Y Wang Y Wang F Zhang J Li S. Transcutaneous auricular vagus nerve stimulators: a review of past, present, and future devices. Expert Rev Med Devices. (2022) 19:43–61. doi: 10.1080/17434440.2022.2020095, PMID: 34937487

[4] Yu Y Yang Y Wang L Fang J Chen Y He JH. Transcutaneous auricular vagus nerve stimulation in disorders of consciousness monitored by fMRI: the first case report. Brain Stimul. (2017) 10:328–30. doi: 10.1016/j.brs.2016.12.004, PMID: 28017322

[5] Briand M-M Gosseries O Staumont B Laureys S Thibaut A. Transcutaneous auricular vagal nerve stimulation and disorders of consciousness: a hypothesis for mechanisms of action. Front Neurol. (2020) 11:933. doi: 10.3389/fneur.2020.00933, PMID: 32982941 PMC7477388

[6] Osińska A Rynkiewicz A Binder M Komendziński T Borowicz A Leszczyński A. Non-invasive Vagus nerve stimulation in treatment of disorders of consciousness – longitudinal case study. Front Neurosci. (2022) 16:834507. doi: 10.3389/fnins.2022.834507, PMID: 35600632 PMC9120963

[7] Noé E Ferri J Colomer C Moliner B O'Valle M Ugart P. Feasibility, safety and efficacy of transauricular vagus nerve stimulation in a cohort of patients with disorders of consciousness. Brain Stimul. (2020) 13:427–9. doi: 10.1016/j.brs.2019.12.005, PMID: 31866491

[8] Yu Y Yang Y Gan S Guo S Fang J Wang S. Cerebral hemodynamic correlates of transcutaneous auricular vagal nerve stimulation in consciousness restoration: an open-label pilot study. Front Neurol. (2021) 12:684791. doi: 10.3389/fneur.2021.684791, PMID: 34335449 PMC8319239

[9] Wang Y Yang Y Wang Y Zhang J. Transcutaneous auricular vague nerve stimulation improved brain connection activity on patients of disorders of consciousness: a pilot study. J Tradit Chin Med. (2022) 42:463–71. doi: 10.19852/j.cnki.jtcm.2022.03.012, PMID: 35610018 PMC9924658

[10] Gianlorenco ACL de Melo PS Marduy A Kim AY Kim CK Choi H. Electroencephalographic patterns in taVNS: a systematic review. Biomedicine. (2022) 10:2208. doi: 10.3390/biomedicines10092208, PMID: 36140309 PMC9496216

[11] Mashour GA Roelfsema P Changeux J-P Dehaene S. Conscious processing and the global neuronal workspace hypothesis. Neuron. (2020) 105:776–98. doi: 10.1016/j.neuron.2020.01.026, PMID: 32135090 PMC8770991

[12] Schiff N. Mesocircuit mechanisms in the diagnosis and treatment of disorders of consciousness. Presse Med. (2022) 52:104161. doi: 10.1016/j.lpm.2022.104161 36563999

[13] Xia X Yang Y Guo Y Bai Y Dang Y Xu R. Current status of Neuromodulatory therapies for disorders of consciousness. Neurosci Bull. (2018) 34:615–25. doi: 10.1007/s12264-018-0244-4, PMID: 29916112 PMC6060218

[14] Rahman A Reato D Arlotti M Gasca F Datta A Parra LC. Cellular effects of acute direct current stimulation: somatic and synaptic terminal effects: somatic and terminal origin of DCS effects. J Physiol. (2013) 591:2563–78. doi: 10.1113/jphysiol.2012.247171, PMID: 23478132 PMC3678043

[15] Huang W Wannez S Fregni F Hu X Jing S Martens G. Repeated stimulation of the posterior parietal cortex in patients in minimally conscious state: a sham-controlled randomized clinical trial. Brain Stimul. (2017) 10:718–20. doi: 10.1016/j.brs.2017.02.001, PMID: 28259543

[16] Santos Ferreira I Teixeira Costa B Lima Ramos C Lucena P Thibaut A Fregni F. Searching for the optimal tDCS target for motor rehabilitation. J NeuroEng Rehabil. (2019) 16:90. doi: 10.1186/s12984-019-0561-5, PMID: 31315679 PMC6637619

[17] Zhang X Liu B Li Y Duan G Hou J Wu D. Multi-target and multi-session transcranial direct current stimulation in patients with prolonged disorders of consciousness: a controlled study. Front Neurosci. (2021) 15:641951. doi: 10.3389/fnins.2021.641951, PMID: 34566555 PMC8456025

[18] Gusnard DA Raichle ME. Searching for a baseline: functional imaging and the resting human brain. Nat Rev Neurosci. (2001) 2:685–94. doi: 10.1038/35094500, PMID: 11584306

[19] Luppi AI Craig MM Pappas I Finoia P Williams GB Allanson J. Consciousness-specific dynamic interactions of brain integration and functional diversity. Nat Commun. (2019) 10:4616. doi: 10.1038/s41467-019-12658-9, PMID: 31601811 PMC6787094

[20] Fridman EA Beattie BJ Broft A Laureys S Schiff ND. Regional cerebral metabolic patterns demonstrate the role of anterior forebrain mesocircuit dysfunction in the severely injured brain. Proc Natl Acad Sci U S A. (2014) 111:6473–8. doi: 10.1073/pnas.1320969111, PMID: 24733913 PMC4035959

[21] Guo Y Bai Y Xia X Li J Wang X Dai Y. Effects of long-lasting high-definition transcranial direct current stimulation in chronic disorders of consciousness: a pilot study. Front Neurosci. (2019) 13:412. doi: 10.3389/fnins.2019.00412, PMID: 31114475 PMC6502996

[22] Guo Y Li R Zhang R Liu C Zhang L Zhao D. Dynamic changes of brain activity in patients with disorders of consciousness during recovery of consciousness. Front Neurosci. (2022) 16:878203. doi: 10.3389/fnins.2022.878203, PMID: 35720697 PMC9201077

[23] Han J Chen C Zheng S Zhou T Hu S Yan X. Functional connectivity increases in response to high-definition transcranial direct current stimulation in patients with chronic disorder of consciousness. Brain Sci. (2022) 12:1095. doi: 10.3390/brainsci12081095, PMID: 36009158 PMC9405975

[24] Jang SH Cho MJ. Transcutaneous auricular vagus nerve stimulation in disorders of consciousness: a mini-narrative review. Medicine. (2022) 101:e31808. doi: 10.1097/MD.0000000000031808, PMID: 36550876 PMC9771208

[25] Ashizuka A Mima T Sawamoto N Aso T Oishi N Sugihara G. Functional relevance of the precuneus in verbal politeness. Neurosci Res. (2015) 91:48–56. doi: 10.1016/j.neures.2014.10.009, PMID: 25455744

[26] Huang Y Datta A Bikson M Parra LC. (2018). ROAST: an open-source, fully-automated, realistic volumetric-approach-based simulator for TES. In: 40th annual international conference of the IEEE engineering in medicine and biology society (EMBC). Honolulu, HI: IEEE (2018). p. 3072–3075. 10.1109/EMBC.2018.8513086 30441043

[27] Schnakers C Chatelle C Vanhaudenhuyse A Majerus S Ledoux D Boly M. The nociception coma scale: a new tool to assess nociception in disorders of consciousness. Pain. (2010) 148:215–9. doi: 10.1016/j.pain.2009.09.028, PMID: 19854576

[28] Chatelle C Majerus S Whyte J Laureys S Schnakers C. A sensitive scale to assess nociceptive pain in patients with disorders of consciousness. J Neurol Neurosurg Psychiatry. (2012) 83:1233–7. doi: 10.1136/jnnp-2012-302987, PMID: 22906615

[29] Lehmann D Skrandies W. Reference-free identification of components of checkerboard-evoked multichannel potential fields. Electroencephalogr Clin Neurophysiol. (1980) 48:609–21. doi: 10.1016/0013-4694(80)90419-8, PMID: 6155251

[30] Murray MM Brunet D Michel CM. Topographic ERP analyses: a step-by-step tutorial review. Brain Topogr. (2008) 20:249–64. doi: 10.1007/s10548-008-0054-5, PMID: 18347966

[31] Custo A Van De Ville D Wells WM Tomescu MI Brunet D Michel CM. Electroencephalographic resting-state networks: source localization of microstates. Brain Connect. (2017) 7:671–82. doi: 10.1089/brain.2016.0476, PMID: 28938855 PMC5736178

[32] Krone L Frase L Piosczyk H Selhausen P Zittel S Jahn F. Top-down control of arousal and sleep: fundamentals and clinical implications. Sleep Med Rev. (2017) 31:17–24. doi: 10.1016/j.smrv.2015.12.005 26883160

[33] Cain JA Spivak NM Coetzee JP Crone JS Johnson MA Lutkenhoff ES. Ultrasonic deep brain neuromodulation in acute disorders of consciousness: a proof-of-concept. Brain Sci. (2022) 12:428. doi: 10.3390/brainsci12040428, PMID: 35447960 PMC9032970

[34] Thibaut A Schiff N Giacino J Laureys S Gosseries O. Therapeutic interventions in patients with prolonged disorders of consciousness. Lancet Neurol. (2019) 18:600–14. doi: 10.1016/S1474-4422(19)30031-6 31003899

[35] Feng Y Zhang J Zhou Y Bai Z Yin Y. Noninvasive brain stimulation for patients with a disorder of consciousness: a systematic review and meta-analysis. Rev Neurosci. (2020) 31:905–14. doi: 10.1515/revneuro-2020-0033, PMID: 32845870

[36] Bender Pape TL Herrold AA Livengood SL Guernon A Weaver JA Higgins JP. A pilot trial examining the merits of combining amantadine and repetitive transcranial magnetic stimulation as an intervention for persons with disordered consciousness after TBI. J Head Trauma Rehabil. (2020) 35:371–87. doi: 10.1097/HTR.0000000000000634, PMID: 33165151

[37] Lin Y Liu T Huang Q Su Y Chen W Gao D. Electroencephalography and functional magnetic resonance imaging-guided simultaneous transcranial direct current stimulation and repetitive transcranial magnetic stimulation in a patient with minimally conscious state. Front Neurosci. (2019) 13:746. doi: 10.3389/fnins.2019.00746, PMID: 31417339 PMC6685103

[38] Sun JB Tian QQ Yang XJ Deng H Li N Meng LX. Synergistic effects of simultaneous transcranial direct current stimulation (tDCS) and transcutaneous auricular vagus nerve stimulation (taVNS) on the brain responses. Brain Stimul. (2021) 14:417–9. doi: 10.1016/j.brs.2021.02.010, PMID: 33621676

[39] Amiri M Fisher PM Raimondo F Sidaros A Cacic Hribljan M Othman MH. Multimodal prediction of residual consciousness in the intensive care unit: the CONNECT-ME study. Brain. (2023) 146:50–64. doi: 10.1093/brain/awac335, PMID: 36097353 PMC9825454

[40] Bai Y He J Xia X Wang Y Yang Y di H. Spontaneous transient brain states in EEG source space in disorders of consciousness. NeuroImage. (2021) 240:118407. doi: 10.1016/j.neuroimage.2021.118407, PMID: 34280527

[41] Lemaire JJ Pontier B Chaix R El Ouadih Y Khalil T Sinardet D. Neural correlates of consciousness and related disorders: from phenotypic descriptors of behavioral and relative consciousness to cortico-subcortical circuitry. Neurochirurgie. (2022) 68:212–22. doi: 10.1016/j.neuchi.2021.05.003, PMID: 34051246

[42] Huang Y Mohan A McLeod SL Luckey AM Hart J Vanneste S. Polarity-specific high-definition transcranial direct current stimulation of the anterior and posterior default mode network improves remote memory retrieval. Brain Stimul. (2021) 14:1005–14. doi: 10.1016/j.brs.2021.06.007, PMID: 34182233

[43] Zhang C Han S Li Z Wang X Lv C Zou X. Multidimensional assessment of electroencephalography in the neuromodulation of disorders of consciousness. Front Neurosci. (2022) 16:903703. doi: 10.3389/fnins.2022.903703, PMID: 35812212 PMC9260110

[44] Zhang R Zhang L Guo Y Shi L Gao J Wang X. Effects of high-definition transcranial direct-current stimulation on resting-state functional connectivity in patients with disorders of consciousness. Front Hum Neurosci. (2020) 14:560586. doi: 10.3389/fnhum.2020.560586, PMID: 33100996 PMC7546763

[45] Xu Z Zheng R Xia T Qi Z Zang D Wang Z. Behavioral effects in disorders of consciousness following transcranial direct current stimulation: a systematic review and individual patient data meta-analysis of randomized clinical trials. Front Neurol. (2022) 13:940361. doi: 10.3389/fneur.2022.940361, PMID: 36247787 PMC9558708

[46] Russo C Souza Carneiro MI Bolognini N Fregni F. Safety review of transcranial direct current stimulation in stroke. Neuromodulation: Technology at the Neural. Interface. (2017) 20:215–22. doi: 10.1111/ner.12574, PMID: 28220641 PMC5389927

[47] Kim AY Marduy A De Melo PS Gianlorenco AC Kim CK Choi H. Safety of transcutaneous auricular vagus nerve stimulation (taVNS): a systematic review and meta-analysis. Sci Rep. (2022) 12:22055. doi: 10.1038/s41598-022-25864-1 36543841 PMC9772204

[48] Bikson M Grossman P Thomas C Zannou AL Jiang J Adnan T. Safety of transcranial direct current stimulation: Evidence based update. Brain Stimul. (2016) 9:641–61. doi: 10.1016/j.brs.2016.06.004 27372845 PMC5007190

[49] Liu Z Si L Xu W Zhang K Wang Q Chen B. Characteristics of EEG microstate sequences during propofol-induced alterations of brain consciousness states. IEEE Trans Neural Syst Rehabil Eng. (2022) 30:1631–41. doi: 10.1109/TNSRE.2022.3182705, PMID: 35696466

[50] Michel CM Koenig T. EEG microstates as a tool for studying the temporal dynamics of whole-brain neuronal networks: a review. NeuroImage. (2018) 180:577–93. doi: 10.1016/j.neuroimage.2017.11.062 29196270

[51] Stefan S Schorr B Lopez-Rolon A Kolassa I-T Shock JP Rosenfelder M. Consciousness indexing and outcome prediction with resting-state EEG in severe disorders of consciousness. Brain Topogr. (2018) 31:848–62. doi: 10.1007/s10548-018-0643-x 29666960

[52] Xu J Pan Y Zhou S Zou G Liu J Su Z. EEG microstates are correlated with brain functional networks during slow-wave sleep. NeuroImage. (2020) 215:116786. doi: 10.1016/j.neuroimage.2020.116786, PMID: 32276057

[53] Gui P Jiang Y Zang D Qi Z Tan J Tanigawa H. Assessing the depth of language processing in patients with disorders of consciousness. Nat Neurosci. (2020) 23:761–70. doi: 10.1038/s41593-020-0639-1, PMID: 32451482

